# 599. Rapid Molecular Identification of *Staphylococcus aureus* and Methicillin Resistance in Blood Isolates Leads to Improved Patient Outcomes

**DOI:** 10.1093/ofid/ofad500.666

**Published:** 2023-11-27

**Authors:** Cecilia Volk, Ethan Meyer, Deanna Jacobs, Warren Rose

**Affiliations:** University of Wisconsin-Madison, Madison, Wisconsin; University of Wisconsin-Madison, School of Pharmacy, Madison, Wisconsin; University of Wisconsin- Madison, School of Pharmacy, Madison, Wisconsin; University of Wisconsin-Madison, Madison, Wisconsin

## Abstract

**Background:**

*Staphylococcus aureus* bacteremia (SaB) is a common infection with a high mortality rate. Optimal therapy for SaB depends on susceptibility, and the use of anti-staphylococcal beta-lactams (ASBL) for MSSA bacteremia leads to lower mortality rates compared to vancomycin. For this reason, rapid susceptibility results, such as molecular detection using the Cepheid Xpert MRSA/SA Blood Culture PCR system, are important for patient management. At our institution, providers have varying levels of confidence in this assay, with some preferring to wait for traditional susceptibility results (AST) before narrowing therapy. This study aimed to both assess concordance between PCR results and AST and determine if waiting for AST leads to differences in patient outcomes.

**Methods:**

All patients diagnosed with SaB at a single US academic medical center during 2019-2022 were included in a retrospective analysis. Data regarding blood culture results, administered antibiotics, and all-cause mortality was collected. For MSSA, optimized therapy was defined as an ASBL (oxacillin or cefazolin).

**Results:**

A total of 288 patients with SaB were included (22.2% MRSA). Three isolates had discordant PCR and AST, all reported an MSSA isolate as MRSA. For MRSA, the positive-predictive value was 95.5% and negative-predictive value was 100%. The average time between PCR and AST results was 38.3 hours. Amongst 220 patients with MSSA bacteremia, 28 never received optimized therapy due to early mortality or other infections/conditions. Of the remaining 192 patients, 137 started an ASBL based on PCR results. The average time from culture draw to optimized therapy was 28.9 hours. Fifty-five patients did not receive ASBL until after AST was available, which increased the time to optimized therapy to 72.7 hours. This delay in therapy led to a significant increase in 30-day mortality rate (21.8% vs 10.2%, P=0.0337).

**Conclusion:**

The Cepheid Xpert MRSA/SA Blood Culture had reliable results that led to a faster time to optimized therapy. Lower mortality was observed when providers opted to start an ASBL based on MSSA PCR results instead of waiting for AST. The mortality benefit of rapid PCR testing in SaB appears to stem from earlier initiation of ASBL therapy for MSSA in addition to improving optimization of MRSA therapies.

**Disclosures:**

**Warren Rose, PharmD, MPH**, Basilea: Honoraria|Ferring: Honoraria|Merck: Grant/Research Support|Paratek: Grant/Research Support|Pfizer: Honoraria

